# Reply to: Arguments for a comet as cause of the Hopewell airburst are unsubstantiated

**DOI:** 10.1038/s41598-022-16212-4

**Published:** 2022-07-15

**Authors:** Kenneth Barnett Tankersley, Stephen D. Meyers, Stephanie A. Meyers, David L. Lentz

**Affiliations:** 1 Department of Anthropology, University of Cincinnati, Cincinnati, OH 45221 USA; 2 Department of Geology, University of Cincinnati, Cincinnati, OH 45221 USA; 3 Department of Biology, University of Cincinnati, Cincinnati, OH 45221 USA

**Keywords:** Environmental social sciences, Astronomy and planetary science

**replying to**: R. Neuhäuser and D. L. Neuhäuser; *Scientific Reports* 10.1038/s41598-022-16211-5 (2022).

## Introduction

We would like to thank Neuhäuser and Neuhäuser^1^ for their critical review of our paper^2^. We presented multi-proxy evidence of an airburst event, which occurred in the Ohio River valley 1699–1567 years ago (252–383 CE). Support for the occurrence of an airburst event includes a disruption in vegetation, meteorites, micrometeorites, and positive anomalies of iridium and platinum in radiocarbon dated, charcoal-rich, Hopewell habitation strata. Our suggestion that the airburst was the result of a comet fragment was based on the overlap of proxies from the Ohio River valley and those recovered from KT boundary, YD boundary, and the Tunguska airburst event sites, which have been attributed to the airburst of comet fragments^2–5^.

Neuhäuser and Neuhäuser’s^1^ commentary raises an important question, What proxies are needed to trace the origin of ancient impactors on the Earth? Ancient airburst events from comet fragments and asteroids are difficult to accurately trace. We recognize that asteroids are the parent bodies of chondrites, and they are physically and chemically distinct from comets. Neuhäuser and Neuhäuser^1^ provide substantial theoretical evidence that the Hopewell impactor could not have been a comet fragment. Based on their observations, we concede that the Hopewell airburst was more likely the result of an asteroid exploding in the upper atmosphere, an interpretation, which is more in alignment with current interpretations of the KT boundary, YD boundary, and Tunguska events^6–8^.

**Figure 1:**
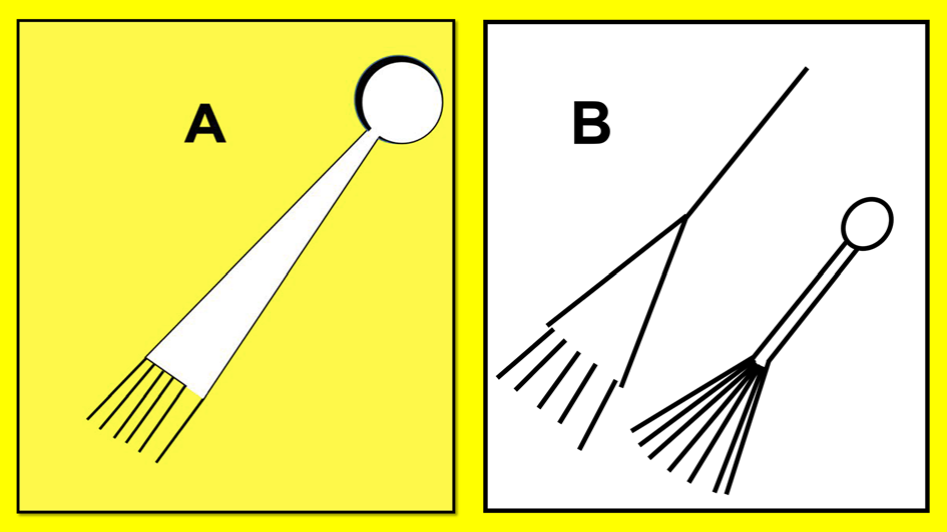
Comparison of a Hopewell earthwork located in near the Hopewell airburst epicenter in Clermont County, Ohio, USA and eyewitness hand drawings of the ~ 12 megaton Tunguska airburst, which occurred on June 30, 1908, in Yeniseysk Governorate, Russia. (**A**) the Milford earthwork; (**B**) assemblage of hand sketches of the Tunguska airburst event^11^. Kenneth Barnett Tankersley created the images using Microsoft PowerPoint for Mac Version 16.41 (www.microsoft.com).

Comparisons drawn with the Tunguska event emphasize the geographic limitations of first-hand observations. The Tunguska airburst was only observed within an 800 km radius of ground zero^9–11^. This fact is significant and demonstrates that major airburst events are not necessarily observed all over the world in northern latitudes. Eyewitness hand drawings of the Tunguska airburst are nearly identical to the Milford Earthwork (Fig. 1). They depict a red ball that “was twice as large as the sun” with “a fiery broom” that “emitted sparks” behind it^12^.

Similarities between the eye witness drawings of the Tunguska airburst and the Milford earthwork are unmistakable. The Milford earthwork is unique. There are no other known Hopewell earthworks like it, which implies that it had special significance to Hopewell people. Earthworks are created by Native Americans as a set of landscape symbols, which reliably convey the same story to any viewer familiar with the symbols^13–15^. Hopewell earthworks are visual representations from which persons versed in the symbolism of the culture can derive a consistent narrative^16^.

The Milford earthwork is in close proximity to the core concentrations of meteorites, positive Pt and Ir anomalies, and micrometeorites. The linear features are not parallel as one would expect of a processional feature: rather, they diverge. The earthwork was originally associated with more conventional earthwork designs, which have been destroyed by development. The airburst portion of the Milford earthwork is located on an elevated terrace, which is 20 m higher than the nearby earthwork enclosed village^17–19^. Our excavation at the Milford earthwork site exposed physical evidence of the Hopewell airburst event in a habitation stratum located beneath an anthropic epipedon, below the level of earthwork construction^2^. Airburst proxies occur in a stratum beneath the earthwork. Based on the law of superposition, the construction of the earthwork post-dates the airburst event horizon.

It is noteworthy that the compass angle of the Milford earthwork is the same compass angle as the geographic distribution of positive Pt and Ir anomalies and the geographic distribution of the maximum size of microspherules. These mutually exclusive proxies support the position that Native Americans accurately portrayed the trajectory of the airburst event. We acknowledge that there may have been supporting details in other near-by earthworks, which since have been destroyed and have gone undocumented.

Neuhäuser and Neuhäuser^1^ objected to our use of Native American oral histories and they generate alternative and varied interpretations for each tradition. Native American symbol systems and oral histories are observations and should not be dismissed as local tales. Rather, they depict an atmospheric phenomenon associated with the airburst. Descendant tribes of the Hopewell have oral histories of a singular event and we are of the opinion that they accurately portray a cosmic airburst.

Native Americans are keen observers of celestial bodies and their relationships with humans and the natural world. Their astronomical knowledge has been passed down over thousands of years as oral histories and symbolically represented in art, communal ceremonies, dance, songs, storytelling, and rituals^13–15^. Different Native American tribes have different names for the same symbolic creature (Cherokee-*Uktenah*, Haudenosaunee-*Dajoji*, Myaamia-*Lenipinšia*, Shawnee-*Tekoomsē*), a horned serpent known as the Sky Panther, a creature that appears at the time of cosmic chaos^20^.

**Figure 2:**
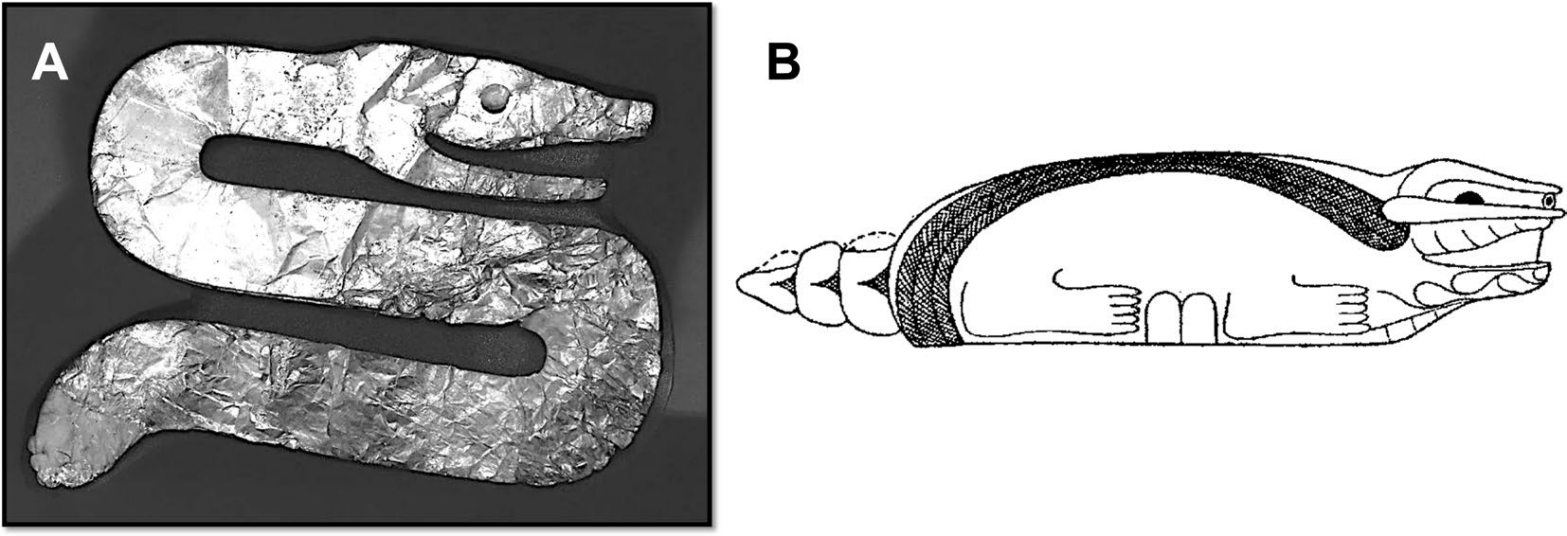
Sky Panthers, also known as horned serpents, excavated in direct association with a pallasite from a Hopewell archaeological feature known as “altar 4” at the Turner site, located near the epicenter of the airburst in Hamilton County, Ohio, USA. (**A**) Hopewell Sky Panther or horned serpent carved from mica; (**B**) Hopewell Sky Panther or horned serpent manufactured from ground-stone. Kenneth Barnett Tankersley created the figure using Microsoft PowerPoint for Mac Version 16.41 (www.microsoft.com).

Neuhäuser and Neuhäuser^1^ state that *Tekoomsē* is a reference to “shooting stars” rather than a comet. They use a non-Native American spelling of *Tekoomsē* (i.e., Tecumseh) and state that there are multiple intepretations of his name*.* Changes in Native American names occur with changes in a person’s life^20^. In Algonquian, *Tekoomsē* means “blazing comet”^21^. The name *Tekoomsē*, Sky Panther, was given in 1769, within the first year of his life, when comet C/1769 P1 was visible to the naked eye^21^. It is also noteworthy that comet C/1811 F1 is known as “Tecumseh's Comet”^22^. *Tekoomsē* believed the comet was a good sign for intertribal unification^21,22^. We also find it significant that multiple Hopewell images of the Sky Panther (horned serpent) were found at the epicenter of the airburst and in direct association with a pallasite (Fig. 2)^20,23^.

Neuhäuser and Neuhäuser^1^ raised the issue that Hopewell people traded meteorites. This suggestion was first positied in 1882 by Charles Louis Metz and Frederick Ward Putnam^20,23^. In 1882, Metz found the first pallasites at the Turner site, and that same year, the Brenham pallasite strewn field was discovered in Kiowa County, Kansas^24^. Because pallasites are a rare form of stony-iron meteorites, and they comprise a very small percentage of all meteorites, Metz and Putnuam presumed that the Turner site specimens had been collected from the recently discovered Brenham strewn field^20,24^.

Native Americans have traded exotic materials across more than 2000 km since the late Pleistocene^25^. Octahedrites and pallasites contain ductile and malleable nickel and iron. Thus, we would expect meteorites to have been valuable trade items throughout the region for more than 15,000 years. Instead, we find meteorites temporally restricted to the Hopewell cultural complex, ~ 250–380 CE. Furthermore, pallasites < 2.5 mm in diameter from the epicenter of the airburst cannot be explained as trade items^2^. Also, the concentrations of Ga and Ge in the Ohio River valley pallasites are 10% lower than the Brenham pallasites and Pt is five times lower^2^.

Neuhäuser and Neuhäuser^1^ suggest that the Hopewell airburst could have been produced by a small meteorite. However, a meteoritic airburst cannot explain the co-occurrence of both octahedrites and pallasites. Likewise, it does not explain the descendant Native American oral histories and earthwork symbolism. There will always be some degree of ambiguity in explaining the cause of ancient airburst events and tracing the origin of ancient impactors on the Earth. While we agree with Neuhäuser and Neuhäuser's^1^ arguments that the Hopewell airburst event was likely the result of an asteroid rather than a comet, their suggestion of a meteoritic airburst is not persuasive.

## Data Availability

No datasets were generated or analyzed during the current study, others than those published in Tankersley et al.^2^.
